# A Recombinant Collagen–mRNA Platform for Controllable Protein Synthesis

**DOI:** 10.1002/cbic.201500205

**Published:** 2015-05-26

**Authors:** Liping Sun, Yunjing Xiong, Anat Bashan, Ella Zimmerman, Shirley Shulman Daube, Yoav Peleg, Shira Albeck, Tamar Unger, Hagith Yonath, Miri Krupkin, Donna Matzov, Ada Yonath

**Affiliations:** [a] Department of Biomaterials, College of MaterialsXiamen University, 422, Siming South Road, Xiamen 361005 (China); [b] Weizmann Institute of Science, 234 Herzl StreetRehovot 7610001 (Israel); [c] Sheba Medical Center, 1 Sheba StreetTel Hashomer 52621 (Israel); [d] Sackler School of Medicine, Tel Aviv University10 Levanon Street, Tel Aviv 69978 (Israel)

**Keywords:** collagen, mRNA, protein synthesis, ribosomes

## Abstract

We have developed a collagen–mRNA platform for controllable protein production that is intended to be less prone to the problems associated with commonly used mRNA therapy as well as with collagen skin-healing procedures. A collagen mimic was constructed according to a recombinant method and was used as scaffold for translating mRNA chains into proteins. Cysteines were genetically inserted into the collagen chain at positions allowing efficient ribosome translation activity while minimizing mRNA misfolding and degradation. Enhanced green fluorescence protein (eGFP) mRNA bound to collagen was successfully translated by cell-free *Escherichia coli* ribosomes. This system enabled an accurate control of specific protein synthesis by monitoring expression time and level. Luciferase–mRNA was also translated on collagen scaffold by eukaryotic cell extracts. Thus we have demonstrated the feasibility of controllable protein synthesis on collagen scaffolds by ribosomal machinery.

Local protein deficiency is a main cause of medical problems ranging from local injury, wounds or surgery that can lead to death to disability or genetic disorders such as cystic fibrosis (CF).[1] Direct injection of the relevant proteins into the tissue might require repeated injections that could cause severe side effects, including initial burst release.[2] Other strategies for tissue regeneration include cell transplantation and gene therapy. A prominent problem associated with autologous cells implants is the difficulty in harvesting a sufficient amount of cells. Moreover, allogeneic and xenogeneic cells possess risks of viral infection and can be immunogenic. Similarly, gene therapy with DNA plasmids or viral vectors has potential disadvantages, including poor control of dosage and duration, low gene-transfer efficiency, endogenous virus recombination, oncogenic effects, and unexpected immune responses.[3] Thus, an efficient local protein synthesis platform for wound areas, deficient tissues or damaged organs should help regain proper functionality as well as promote tissue repair.

Ribosomes are the universal molecular machines that perform protein biosynthesis according to the genetic code provided to them by messenger RNA (mRNA). So far, in vivo on-site-induced protein biosynthesis by ribosomes has hardly been attempted. Nanoparticles encapsulating luciferase-encoding plasmid and *Escherichia coli* extract are capable of autonomous synthesis of protein in vitro and in vivo.[4] mRNA vectors have also been used as templates for protein synthesis rather than DNA plasmid due to RNA's low toxicity, low immunogenicity, and high expression efficacy in vivo. It has been shown that modified RNA encoding human vascular endothelial growth factor-A (VEGF-A) led to marked improvement in heart function in a mouse myocardial infarction model.[5]

Collagen, which is widely used for tissue engineering, is the main structural protein of various connective tissues.[6] All collagen molecules are made up of three polypeptide strands, twisted together into a right-handed triple helix. A distinctive feature of collagen is the repeated GXY sequence, in which G represents glycine and X and Y may be any amino acid. The glycines are located in the interior of the helix, and the amino acids at the Y positions are located at the helix surface.[7] Hence their side chains may be chemically modified without perturbing the stable helical structure (Figure S1 in the Supporting Information). Natural collagen can suffer from contamination by infectious agents, heterogeneity, potential immunogenicity, loss of structural integrity, and product standardization.[8] An efficient recombinant collagen should be free of most of these complications, as this is less immunogenic and offers excellent homogeneity. More importantly, collagen gene sequence can be designed according to specific requirements.[9]

The use of immobilized mRNA has been reported previously. Solid surfaces, that is, chip surfaces coated with neutravidin were used for smFRET studies[10] and streptavidin-coated beads were useful for other applications.[11] However, such systems are less suitable for in situ tissue repair, which requires soft carriers. Hence, we focus on mRNA immobilization on natural biological scaffolds, such as collagen.

Benefiting from knowledge of the accurate ribosomal machinery[12] alongside collagen's chemical and structural properties,[7] we have developed a new, controllable, recombinant collagen–mRNA system for local protein production. We designed and cloned a collagen gene into which cysteine codons were inserted at positions allowing efficient mRNA binding and ribosome translation activity (Figure S2). As natural collagen contains no cysteine in the GXY repeats region, we inserted two glycine-proline-cysteine (GPC) triplets into our recombinant collagen to provide a functional sulfhydryl group for further reaction with amine-modified mRNA. A bacteriophage T4 fibritin foldon domain at the C terminus serves as a nucleation site to facilitate the correct folding of the collagen triple helix.[13]

Enhanced green fluorescence protein (eGFP) was used as a reporter protein to investigate the feasibility of controllable protein synthesis on collagen scaffold by the ribosomal machinery. mRNA of eGFP was crosslinked to the collagen strand, and eGFP was translated when ribosomes were provided by *E. coli* extract. Thus, here we demonstrate the construction of a new recombinant collagen–mRNA platform for controllable continuous specific protein synthesis.

Our construct contains collagen bound to an N-terminal maltose binding protein (MBP) domain, a hexahistidine tag, a TEV cleavage site, a Flag tag, and a foldon domain at the C terminus (Figure 1). In principle, our construct could be of a large range of lengths (repeats of the collagen portion), according to need.

**Figure 1:**
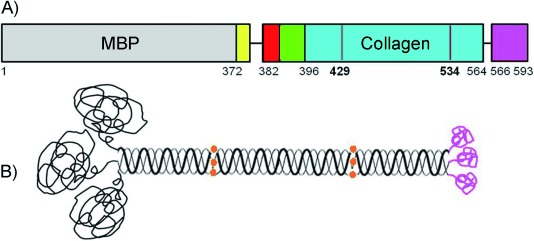
A) Schematic representation of the recombinant MBP–collagen. The domains are not drawn to scale; numbers show amino acid positions, with the cysteine residues marked in bold. Yellow, red, green, and purple represent the His_6_-tag, TEV cleavage site, Flag-tag, and foldon domain, respectively. B) Triple-helix representation of MBP–collagen. Left: N-terminal folded MBP domains are (black), right C-terminal foldon domains (purple). Orange spheres represent cysteines in collagen strands.

The tunable distance between the inserted cysteines can be designed to maximize the efficiency of the platform, which is designed such that 1) the SH= insertions should minimize mRNA aggregation and/or misfolding and 2) the platform's multiple sizes are fully under control. This distance, which controls the usability of the platforms (as it directly influences the possible interactions between the bound mRNA chains), depends on the structure of the recombinant modified collagen scaffold. Hence we verified that it maintains the triple helicity of natural collagen (see below).

An additional parameter is the ribosome size, which is different in prokaryotes and eukaryotes. In this study it was set to 28.8 nm (105 amino acids of the collagen chain, after verifying the formation of triple helices), which is sufficient for *E. coli* ribosome binding; as its longest dimension is about 25 nm. The chimeric protein MBP–collagen (Figure S2) was expressed in *E. coli* BL21(DE3) cells and was found to fold as a trimer with a calculated molecular weight (MW) of 184.5 kDa (Figure 2 A, lane 1). After 5 min of heating at 90 °C under reducing conditions, a monomer band appeared (lane 2). These observations were further supported by western-blot analysis with anti His-tag antibody. Lanes 3 and 4 represent trimeric and monomeric MBP–collagen, respectively. These data demonstrated that the collagen trimer was converted to unfolded monomer under reducing condition. Schematic structures of the trimer and monomer are shown in Figure 2 B.

**Figure 2:**
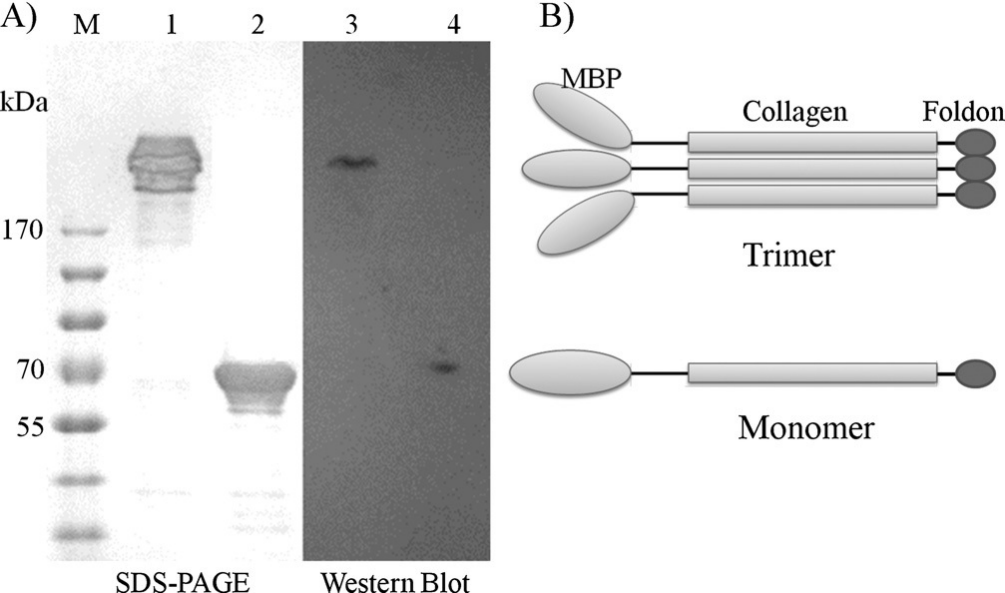
A) SDS-PAGE and Western blot of collagen trimer (unheated sample, lanes 1 and 3) and monomer (heated sample, lanes 2 and 4). B) Schematic representation of the collagen trimer and monomer structures. The calculated molecular weight of the collagen monomer is 61.5 kDa.

The thermostability of MBP–collagen was further studied by circular dichroism (CD) spectroscopy. The typical peaks for a collagen triple helix, namely a negative peak at 196 nm and a positive peak at 224 nm,[14] were observed (Figure 3 A). When a sample was heated from 20 to 90 °C, the heights of these peaks decreased gradually, thereby indicating denaturation of the collagen. The melting temperature (*T*_m_) was around 55 °C (Figure 3 B). Given the *T*_m_ of human type I collagen (<36 °C), the recombinant MBP–collagen is highly stable. This thermal stability is attributed to the foldon domain and the multiple GPP repeats in MBP–collagen.[10] We also investigated the thermostability of MBP–collagen by SDS-PAGE (Figure 3 C). MBP–collagen samples were incubated at different temperatures (30–90 °C) for 5 min and analyzed by 10 % SDS-PAGE. We found that melting took place at 50 °C, in accordance with the CD result.

**Figure 3:**
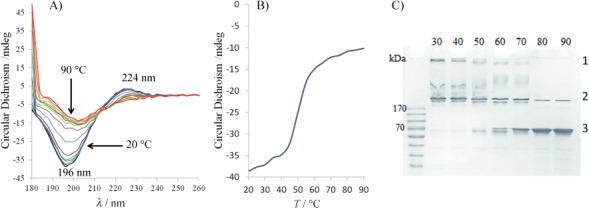
A) CD spectra of MBP–collagen taken at 20–90 °C. B) Melting curve of MBP–collagen at 196 nm. C) SDS-PAGE of MBP–collagen at 30–90 °C. The positions of the loading well, trimer, and monomer are marked 1, 2 and 3, respectively.

We designed a protein synthesis platform in which mRNA was covalently bound to a collagen scaffold. In this system, the 3′-end of the desired mRNA is modified with an amine and crosslinked to engineered cysteines on MBP–collagen by using the hetero-bifunctional crosslinker sulfo-SMCC.[15] Crosslinking the mRNA through its 3′-end rather than the 5′ might increase the accessibility of the 5′-end of the mRNA to ribosomes.

A titration experiment was performed to determine the optimal molar ratio of collagen to RNA. Accordingly, crosslinking was performed at a 20:1 collagen:mRNA ratio in order to ensure that no free RNA remained in the solution (Figure 4 A). The products of the two-step crosslinking reaction were resolved by SDS denaturing agarose gel electrophoresis (Figure 4 B). Nevertheless, it is conceivable that part of the mRNA was nonspecifically bound to the collagen. Indeed, we found that under denaturing conditions the collagen–RNA complexes migrate into the gel, providing a comparison between the crosslinking and nonspecific collagen–RNA complexes (Figure 4 B lanes 2 and 3, respectively). The product of the crosslinked reaction migrated slightly faster than the nonspecific complex. In addition, the mRNA was better protected from degradation in the crosslinked reaction, consequently practically no smeared band was observed in the crosslinked RNA–collagen complex (Figure 4 B, lane 2).

**Figure 4:**
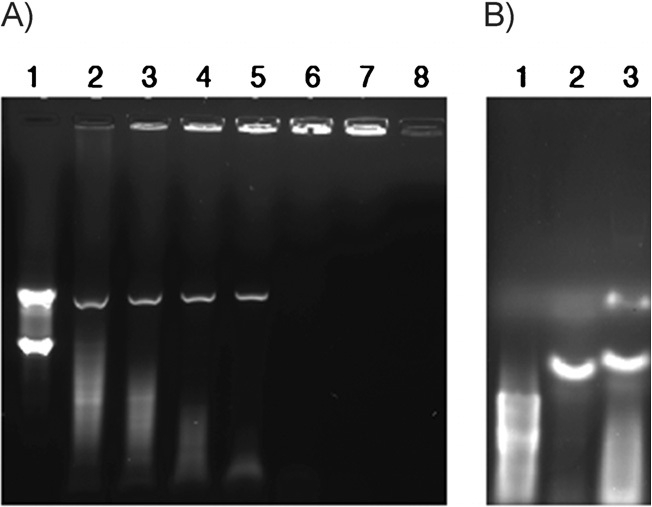
Titration experiment aimed at resolving RNA–collagen complexes by gel electrophoresis. A) Unmodified RNA (lane 1) and collagen (lane 8) were incubated at a final molar ratio of collagen to RNA of 1:1, 2:1, 4:1, 8:1, 16:1 and 32:1 (lanes 2–7, respectively) and resolved on non-denaturing 1 % agarose gel in 0.5 %× TBE buffer. B) Resolution of RNA–collagen complexes by SDS denaturing agarose gel. Complexes formed between unmodified RNA and collagen (lane 3), or modified RNA and crossed linked to collagen (lane 2) were resolved and compared to unmodified RNA only (lane 1). Bands were imaged after ethidium bromide staining.

In order to test whether the mRNA that is part of a collagen–RNA complex could undergo translation by ribosomes, complexes formed in the crosslinking reaction at a 20:1 collagen:RNA ratio were incubated with *E. coli* ribosome extract S12 as the source of ribosomal machinery, energy and ions required for the protein translation.[16] The 20:1 ratio ensured that very little free RNA was present in the reaction (Figure 4 A). We measured the kinetics of eGFP at 37 °C. The relative fluorescence units (RFU) of eGFP increased gradually over time (Figure 5 A), reaching maximal fluorescence intensity after 50 min of incubation; eGFP expression level was also found to be dependent on RNA concentration. The RNA–collagen complex showed a similar kinetic profile to eGFP expression from non-crosslinked RNA (Figure 5 B). These results indicated that eGFP was successfully synthesized while associated with the collagen scaffold.

**Figure 5:**
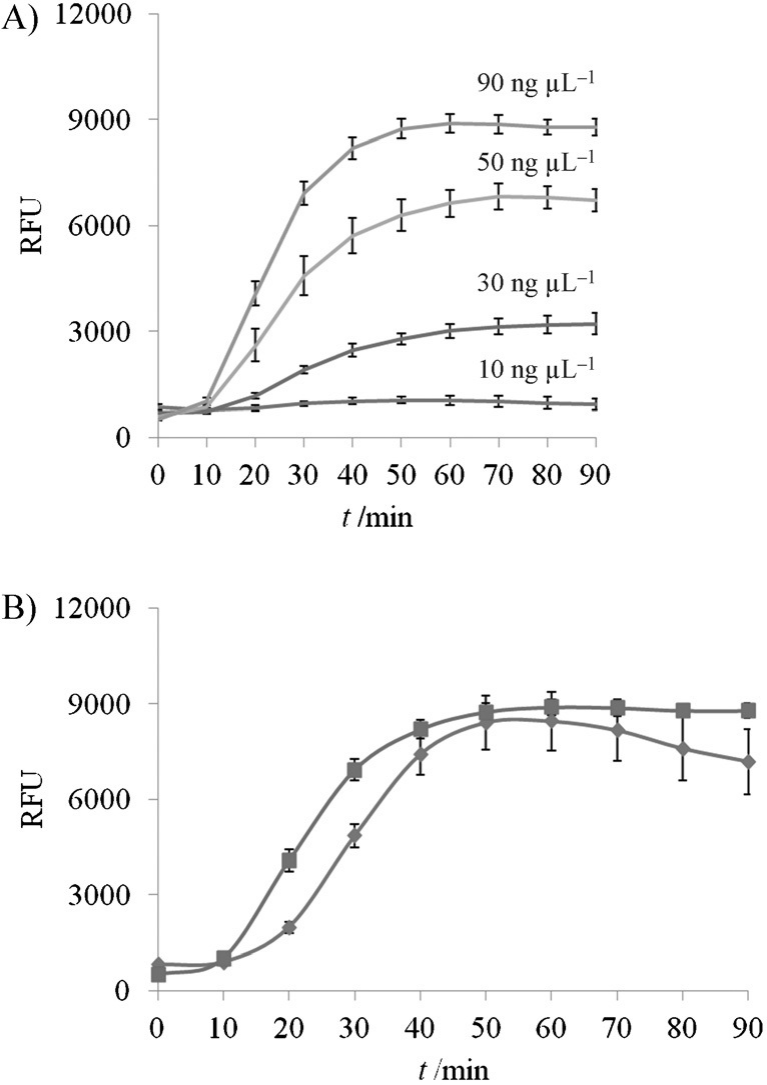
A) Time- and concentration-dependent eGFP biosynthesis on a collagen scaffold ([RNA]=10–90 ng μL^−1^). B) Comparison of eGFP expression of RNA () and RNA-collagen (▪). All results are representative of three repeated experiments. Relative fluorescence units (RFU) represent the means±standard deviations.

To expand the application of the collagen–mRNA platform in eukaryotic systems, we investigated luciferase synthesis on a collagen scaffold with eukaryotic translation extracts (rabbit reticulocyte lysate) instead of *E. coli* ribosome extract. The luciferase mRNA–collagen system showed a significantly higher luminescence intensity (Table S2) than the negative control sample, but a lower one than a pure mRNA system; this might be due to mRNA degradation.

So far mRNA treatment is in its embryonic phase because of issues concerning its stability and its tendency to interact with other mRNA chains, self-fold, or aggregate. Our design should minimize these problems because we can select the location of the mRNA on the collagen platform in a fashion that limits its mobility. One way is to position neighboring mRNA chains at intervals compatible with the size of the ribosome, so that the translating ribosome can occupy the space between them (Figure S3). Nevertheless, insertion of mRNA into cells could lead to side effects such as activating the mammalian innate immune response system; this might be solved by incorporating modified nucleosides in mRNA.[17]

The main advantage of our platform is the controllability of its size, shape, and composition. Thus, the sequences of the mRNA chains can be preselected as well as readily exchanged or modified during treatment according to the medical purpose. Other advantages stem from the properties of the recombinant collagen in the proposed platform. First, its sequence can be designed so as not to include any of the collagen antigenic determinants. Second, we have full control of its size—from very large to relatively small—so that it will be endocytosed by protein-deficient cells. In addition, the concentration of synthesized protein can be controlled by the amount of the cell-free system in local surface applications, such as wounds or by the concentration of the added mRNA platform. Moreover, multiple mRNA chains encoding different proteins can be linked to a single collagen scaffold, or to an assembly of scaffolds, thus enabling a combination therapy of multiple protein and peptide drugs. Consequently, the construct can be used to synthesize diverse functional proteins (e.g., growth factors that can promote tissue repair). As this platform enables accurate control of the amounts of specific proteins by selecting the required mRNA sequence, expression time and expression level, it could also provide a platform for personalized medicine.

## Experimental Section

The collagen gene was optimized for *E. coli* codon usage and cloned into pUC57 plasmid. Collagen was subcloned into a pET-MBP-TevH plasmid by transfer PCR,[18] and the sequence was verified by DNA sequencing. The constructed plasmid was transformed into chemically competent *E. coli* BL21(DE3) cells. The cells were grown at 37 °C in lysogeny broth. Protein expression was induced by isopropyl-d-thiogalactopyranoside (IPTG). MBP–collagen was purified by nickel affinity column chromatography and gel filtration column chromatography on a fast protein liquid chromatography system. SDS-PAGE and western blot were performed to confirm the collagen expression.

EGFP or the luciferase gene was amplified by PCR. PCR products were transcribed to mRNA by T7 RNA polymerase. EGFP RNA was modified with 3′-NH_2_-ATP by using poly-A polymerase. Amine-modified RNA was conjugated to sulfhydryl-containing MBP–collagen by sulfo-SMCC. RNA, collagen and RNA–collagen complex were analyzed by 1 % agarose gel electrophoresis with ethidium bromide staining. EGFP and luciferase syntheses on collagen scaffolds were performed by using an *E. coli* cell-free translation assay and rabbit reticulocyte lysate system, respectively.

S12 cell-free extract was prepared from *E. coli* strain BL21(DE3) according to a previous report.[16] An aqueous solution containing amino acid mix, ATP, *E. coli* tRNA mixture, S12 extract, and other components was prepared. Collagen–mRNA or eGFP–mRNA (250 ng μL^−1^) was added to this solution at 37 °C, thereby commencing the translation reaction. Fluorescence data were collected on a Synergy HT Microplate Reader (BioTek Instruments; *λ*_ex_=485 nm, *λ*_em_=528 nm). Collagen–luciferase mRNA complex or luciferase–mRNA was translated in rabbit reticulocyte lysate system at 30 °C for 1.5 h. Luciferase activity was analyzed by using an Infinite 200 Pro microplate reader (Tecan, Männedorf, Switzerland).

## References

[1] Gadsby DC, Vergani P, Csanády L (2006). Nature.

[2a] Boehler RM, Graham JG, Shea LD (2011). BioTechniques.

[2b] Ikada Y (2006). J. R. Soc. Interface.

[3a] Lee K, Silva EA, Mooney DJ (2011). J. R. Soc. Interface.

[3b] Niidome T, Huang L (2002). Gene Ther.

[4] Schroeder A, Goldberg MS, Kastrup C, Levins CG, Langer R, Anderson DG (2012). NanoLetters.

[5] Zangi L, Lui KO, Gise A, Ma Q, Ebina W, Ptaszek LM, Später D, Xu H, Tabebordbar M, Gorbatov R, Sena B, Nahrendorf M, Briscoe DM, Li RA, Wagers AJ, Rossi DJ, Pu WT, Chien KR (2013). Nat. Biotechnol.

[6a] Glowacki J, Mizuno S (2008). Biopolymers.

[6b] Weng B, Liu X, Shepherd R, Wallace GG (2012). Synth. Met.

[7a] Traub W, Yonath A, Segal DM (1969). Nature.

[7b] Yonath A, Traub W (1969). J. Mol. Biol.

[8a] Shoulders MD, Raines RT (2009). Annu. Rev. Biochem.

[8b] Cen L, Liu W, Cui L, Zhang W, Cao Y (2008). Pediatr. Res.

[9a] Liu Y, Griffith M, Watsky MA, Forrester JV, Kuffova L, Grant D, Merrett K, Carlsson DJ (2006). Biomacromolecules.

[9b] Olsen D, Yang C, Bodo M, Chang R, Leigh S, Baez J, Carmichael D, Perala M, Hamalainen ER, Jarvinen M, Polarek J (2003). Adv. Drug Delivery Rev.

[10] Tsai A, Kornberg G, Johansson M, Chen J, Puglisi JD (2014). Cell Rep.

[11] Biyani M, Husimi Y, Nemoto N (2006). Nucleic Acids Res.

[12] Bashan A, Agmon I, Zarivach R, Schluenzen F, Harms J, Berisio R, Bartels H, Franceschi F, Auerbach T, Hansen HAS, Kossoy E, Kessler M, Yonath A (2003). Mol. Cell.

[13] Frank S, Kammerer RA, Mechling D, Schulthess T, Landwehr R, Bann J, Guo Y, Lustig A, Bächinger HP, Engel J (2001). J. Mol. Biol.

[14] Piez KA, Sherman MR (1970). Biochemistry.

[15a] Hermanson GT (2008). Bioconjugate Techniques.

[15b] Hemaprabha E (2012). J. Pharm. Sci. Innov.

[15c] Tannous BA, Chiu NHL, Christopoulos TK (2002). Anal. Chim. Acta.

[15d] B. A. R. Williams, J. C. Chaput, *Curr. Protoc. Nucleic Acid Chem***2010**

[16] Kim TW, Keum JW, Oh IS, Choi CY, Park CG, Kim DM (2006). J. Biotechnol.

[17] Karikó K, Muramatsu H, Welsh FA, Ludwig J, Kato H, Akira S, Weissman D (2008). Mol. Ther.

[18a] Peleg Y, Unger T (2008). Methods in Molecular Biology, Vol. 426: Application of High-Throughput Methodologies to the Expression of Recombinant Proteins in.

[18b] Erijman A, Dantes A, Bernheim R, Shifman JM, Peleg Y (2011). J. Struct. Biol.

