# Supplementary material for: A Recombinant Collagen–mRNA Platform for Controllable Protein Synthesis
Source: Chembiochem. 2015 May 26;16(10):1415–9. doi: 10.1002/cbic.201500205 (PMC4517095; doi:10.1002/cbic.201500205)
Supplement: Supplementary file 1 [file cbic0016-1415-sd1.pdf]

CHEMBIOCHEM

# Supporting Information

## A Recombinant Collagen–mRNA Platform for Controllable Protein Synthesis

Liping Sun,[a] Yunjing Xiong,[a] Anat Bashan,[b] Ella Zimmerman,[b] Shirley Shulman Daube,[b] Yoav Peleg,[b] Shira Albeck,[b] Tamar Unger,[b] Hagith Yonath,[c, d] Miri Krupkin,[b] Donna Matzov,[b] and Ada Yonath*[b]

cbic_201500205_sm_miscellaneous_information.pdf

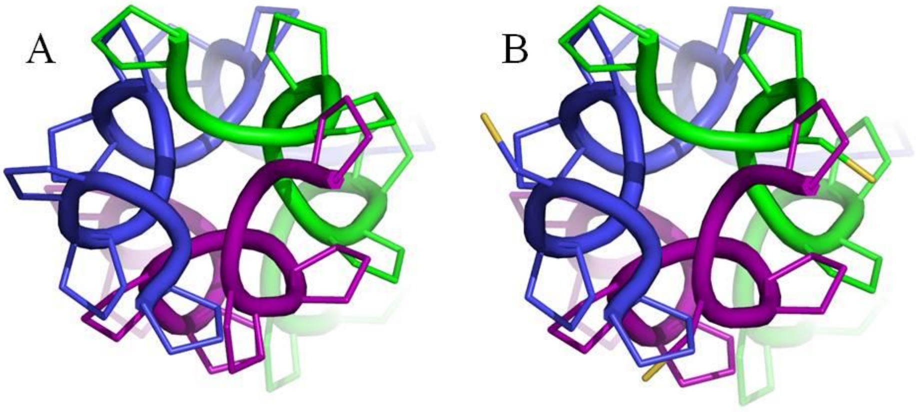


**Figure S1.** Trimeric structure of collagen. Blue, purple and green indicate three α 1 chains, respectively. (A) Glycine-Proline-Proline. (B) Glycine-Proline-Cysteine. The sulfhydryl groups of cysteines are shown as yellow sticks. The figure is made by PyMol[1] using PDB 1K6F and modeling of C instead of P in the original structure.

## I. Cloning of MBP-collagen

Since collagen triple helix is formed in a C- to N-terminal direction and the correct trimerization of the C-terminus is crucial for collagen assembly, the use of C-terminal tags is avoided. We derived the collagen scaffold from a part of human collagen type I, α1 chain (NCBI accession number: NP_000079) with additional 10 Gly-Pro-Pro repeats at both ends for increased stability. MBP domain was fused to the N-terminus to improve the solubility and yield of collagen in *E. coli*. The $his_6$-tag enables purification of MBP-collagen. A bacteriophage T4 fibritin foldon domain at the C terminus serves as a nucleation site to facilitate the correct folding of collagen triple helix.[2]

The following collagen sequence was designed with a His tag (brown) and TEV protease cleavage site (red) at the N terminus. Natural collagen molecule contains no cysteine in the GXY repeats region. To provide the functional sulfhydryl group for further reaction with amine modified mRNA, we inserted two Glycine-Proline-Cysteine (GPC) triplets into our recombinant collagen (underlined).

HHHHHHENLYFQGGPPGPPGPPGPPGPPGPPGPPGPPGPPGPPGPCGSPGRDGSPGAKGDRGETGPAGPPGAPGAPGAPGPV
GPAGKSGDRGETGPAGPAGPVGPVGARGPAGPQGPRGDKGETGEQGDRGIKGHRGFSGLQGPPGPPGPCGPPGPPGPPGPPG
PPGPPGPPGPP GPPGPPGSGYIPEAPRDGQAYVRKDGEWVLLSTFL

Collagen mimic was optimized for *E. coli* codon usage and synthesized with a 5' BamHI and NdeI and 3' SacI followed by a stop codon. It was cloned into pUC57 by GeneWiz (South Plainfield, NJ).

Collagen gene was subcloned into a pET-25b(+) (Novagen) by restriction digest and ligation.

Two separate restriction digests were performed: 5 μ g of collagen-pUC57 plasmid (donor) or 1μ g of pET-25b(+) plasmid (recipient) was digested with SacI and NdeI. The reaction mixture contained 5μ l of 20 U/μ l of each enzyme, 100 μg/ml BSA and 1× NEB buffer 4. The reaction was carried out at room temperature for 2.5-3 h. The Collagen-pUC57 plasmid digest was run on a 1% agarose gel and the bands of collagen gene (651 bp) from the double-cut product were cut out of the gel and purified with MEGA quick-spin™ total fragment DNA purification kit (JH Science, USA). 1 μ l of 10 U/μ l CIP (NEB, USA) was added to 50 μ l of pET-25b(+) plasmid digest and incubated for 30 min at 37 ℃. The reaction mix was treated with 1 μ l CIP again for 30 min at 37 ℃, then purified with MEGA quick-spin™ total fragment DNA purification kit. The ligation of collagen gene into the cut pET-25b(+) plasmid was performed with the ligation mix (Takara, Japan). The reaction mixture for the ligation included approximately 16 ng of cut collagen and 9 ng of pET-25b(+) plasmid digest. The reaction was carried out at room temperature for 30 min, then directly transformed into highly-efficient competent *E. coli* DH5a cells prepared as previously described.[3,4]

Collagen gene was subcloned into a pET-MBP-TevH or pET-GB1-TevH plasmid by transfer-PCR.[5] Primers were ordered from Sigma (Rehovot, Israel). Plasmid purification was carried out using DNA-spin plasmid DNA purification kit (Intron Biotechnology, Daejoen, South Korea).

TPCR conditions were as follows: A single denaturation step (95 ℃, 30 s) followed by 30 cycles of: denaturation (95 ℃, 30 s), annealing (60 ℃, 1 min) and elongation (72 ℃, 5 min) and a final elongation step of 7 min at 72 ℃. TPCR reaction included the following components: 10 ng of the pET-25b(+)-collagen plasmid (donor) and pET-MBP-TevH plasmid (recipient), 20 nM of each forward and reverse primer (TPF and TPR, Table 1), 200 μ M of each dNTP, 1× Phusion buffer and 1.6 U of Phusion DNA polymerase (Finnzymes, Espoo, Finland). After TPCR reaction, 1 μ l of 20 U/μ l DpnI (NEB, USA) was added to 10 μ l of TPCR product followed by incubation for 1–2 h at 37 ℃. The DNA was then directly transformed into competent *E. coli* DH5a cells.

Colony PCR screening was performed using T7 and PetRev primers (Table 1). For such reactions, individual colonies were picked and added to a reaction tube containing 10 μ l Master Mix solution (Ampliqon, Skovlunde, Denmark), 1.25 μ M of each primer and supplemented to a final reaction

volume of 20 μl with water. The colony PCR reaction included a single denaturation step (95 ℃, 1 min) followed by 25 cycles of denaturation (95 ℃, 30 s), annealing (60 ℃, 1 min) and elongation (72 ℃, 1.5 min) and a final elongation step of 6 min at 72 ℃. The PCR reaction products were analyzed on 1% (w/v) agarose gels. For DNA cloning, some of the positive clones were analyzed for the integrity of the DNA sequence of the protein open reading frame, following colony PCR screening. The sequence was verified by DNA sequencing.

**Table S1.** Primers used for cloning and sequencing

| Primer | Function | Sequence (5'-3') |
|---|---|---|
| TPF | MBP-collagen cloning | TCCGCGGGTGAAAACCTGTACTTCCAGGGTGATTATAAAGATGATGACGATAAAGGCC CTCCTGGACCTCCTGG |
| TPR | MBP-collagen cloning | GTGGTGGTGCTCGAGTGCGGCCGCAAGCTTTTACAGGAAGGTACTCAGCAGCAC |
| T7 | Colony PCR, DNA sequencing | ATTAATACGACTCACTATAGGGG |
| PetRev | Colony PCR | ATGCTAGTTATTGCTCAGCGGT |
| MBPF2 | DNA sequencing | ATGTCCGCTTTCTGGTATGCC |
| GFP-F | eGFP PCR | CGTCCGGCGTAGAGGATCGA |
| GFP-R | eGFP PCR | GTTATTGCTCAGCGGTGGCAG |
| Luc-F | luciferase PCR | GTTTTCCCAGTCACGACGTT |
| Luc-R | luciferase PCR | CGAATTCGGATCCCATATGG |

```
  1 ATG AAA ATC GAA GAA GGT AAA CTG GTA ATC TGG ATT AAC GGC GAT AAA GGC TAT AAC GGT
     M   K   I   E   E   G   K   L   V   I   W   I   N   G   D   K   G   Y   N   G  20

 61 CTC GCT GAA GTC GGT AAG AAA TTC GAG AAA GAT ACC GGA ATT AAA GTC ACC GTT GAG CAT
     L   A   E   V   G   K   K   F   E   K   D   T   G   I   K   V   T   V   E   H  40

121 CCG GAT AAA CTG GAA GAG AAA TTC CCA CAG GTT GCG GCA ACT GGC GAT GGC CCT GAC ATT
     P   D   K   L   E   E   K   F   P   Q   V   A   A   T   G   D   G   P   D   I  60

181 ATC TTC TGG GCA CAC GAC CGC TTT GGT GGC TAC GCT CAA TCT GGC CTG TTG GCT GAA ATC
     I   F   W   A   H   D   R   F   G   G   Y   A   Q   S   G   L   L   A   E   I  80

241 ACC CCG GAC AAA GCG TTC CAG GAC AAG CTG TAT CCG TTT ACC TGG GAT GCC GTA CGT TAC
     T   P   D   K   A   F   Q   D   K   L   Y   P   F   T   W   D   A   V   R   Y  100

301 AAC GGC AAG CTG ATT GCT TAC CCG ATC GCT GTT GAA GCG TTA TCG CTG ATT TAT AAC AAA
     N   G   K   L   I   A   Y   P   I   A   V   E   A   L   S   L   I   Y   N   K  120

361 GAT CTG CTG CCG AAC CCG CCA AAA ACC TGG GAA GAG ATC CCG GCG CTG GAT AAA GAA CTG
```

```
     D  L  L  P  N  P  P  K  T  W  E  E  I  P  A  L  D  K  E  L  140

 421 AAA GCG AAA GGT AAG AGC GCG CTG ATG TTC AAC CTG CAA GAA CCG TAC TTC ACC TGG CCG
     K   A   K   G   K   S   A   L   M   F   N   L   Q   E   P   Y   F   T   W   P   160

 481 CTG ATT GCT GCT GAC GGG GGT TAT GCG TTC AAG TAT GAA AAC GGC AAG TAC GAC ATT AAA
     L   I   A   A   D   G   G   Y   A   F   K   Y   E   N   G   K   Y   D   I   K   180

 541 GAC GTG GGC GTG GAT AAC GCT GGC GCG AAA GCG GGT CTG ACC TTC CTG GTT GAC CTG ATT
     D   V   G   V   D   N   A   G   A   K   A   G   L   T   F   L   V   D   L   I   200

 601 AAA AAC AAA CAC ATG AAT GCA GAC ACC GAT TAC TCC ATC GCA GAA GCT GCC TTT AAT AAA
     K   N   K   H   M   N   A   D   T   D   Y   S   I   A   E   A   A   F   N   K   220

 661 GGC GAA ACA GCG ATG ACC ATC AAC GGC CCG TGG GCA TGG TCC AAC ATC GAC ACC AGC AAA
     G   E   T   A   M   T   I   N   G   P   W   A   W   S   N   I   D   T   S   K   240

 721 GTG AAT TAT GGT GTA ACG GTA CTG CCG ACC TTC AAG GGT CAA CCA TCC AAA CCG TTC GTT
     V   N   Y   G   V   T   V   L   P   T   F   K   G   Q   P   S   K   P   F   V   260

 781 GGC GTG CTG AGC GCA GGT ATT AAC GCC GCC AGT CCG AAC AAA GAG CTG GCA AAA GAG TTC
     G   V   L   S   A   G   I   N   A   A   S   P   N   K   E   L   A   K   E   F   280

 841 CTC GAA AAC TAT CTG CTG ACT GAT GAA GGT CTG GAA GCG GTT AAT AAA GAC AAA CCG CTG
     L   E   N   Y   L   L   T   D   E   G   L   E   A   V   N   K   D   K   P   L   300
 901 GGT GCC GTA GCG CTG AAG TCT TAC GAG GAA GAG TTG GCG AAA GAT CCA CGT ATT GCC GCC
     G   A   V   A   L   K   S   Y   E   E   E   L   A   K   D   P   R   I   A   A   320

 961 ACC ATG GAA AAC GCC CAG AAA GGT GAA ATC ATG CCG AAC ATC CCG CAG ATG TCC GCT TTC
     T   M   E   N   A   Q   K   G   E   I   M   P   N   I   P   Q   M   S   A   F   340

1021 TGG TAT GCC GTG CGT ACT GCG GTG ATC AAC GCC GCC AGC GGT CGT CAG ACT GTC GAT GAA
     W   Y   A   V   R   T   A   V   I   N   A   A   S   G   R   Q   T   V   D   E   360

1081 GCC CTG AAA GAC GCG CAG ACT ACT AGT GGT TCT GGT CAT CAC CAT CAC CAT CAC TCC GCG
     A   L   K   D   A   Q   T   T   S   G   S   G   H   H   H   H   H   H   S   A   380

1141 GGT GAA AAC CTG TAC TTC CAG GGT GAT TAT AAA GAT GAT GAC GAT AAA GGC CCT CCT GGA
     G   E   N   L   Y   F   Q   G   D   Y   K   D   D   D   D   K   G   P   P   G   400

1201 CCT CCT GGT CCG CCT GGT CCG CCG GGT CCT CCT GGA CCT CCT GGC CCT CCT GGC CCG CCT
     P   P   G   P   P   G   P   P   G   P   P   G   P   P   G   P   P   G   P   P   420

1261 GGC CCG CCG GGT CCG CCG GGT CCT TGT GGT AGT CCG GGC CGT GAT GGC AGC CCT GGT GCA
```

G P P G P P G P C G S P G R D G S P G A 440

1321 AAA GGC GAC CGC GGT GAA ACA GGC CCT GCC GGT CCT CCG GGT GCC CCG GGT GCA CCT GGC

K G D R G E T G P A G P P G A P G A P G 460

1381 GCA CCT GGT CCG GTT GGC CCT GCC GGT AAG AGC GGT GAT CGC GGC GAA ACA GGT CCT GCC

A P G P V G P A G K S G D R G E T G P A 480

1441 GGC CCG GCC GGT CCT GTG GGT CCT GTT GGT GCA CGC GGT CCG GCA GGT CCG CAA GGT CCT

G P A G P V G P V G A R G P A G P Q G P 500

1501 CGT GGC GAC AAG GGT GAG ACA GGC GAA CAA GGC GAT CGC GGT ATC AAG GGC CAC CGT GGC

R G D K G E T G E Q G D R G I K G H R G 520

1561 TTC AGT GGC CTG CAA GGT CCG CCA GGT CCT CCT GGT CCT TGC GGC CCG CCG GGC CCT CCT

F S G L Q G P P G P P G P C G P P G P P 540

1621 GGC CCT CCT GGT CCT CCA GGT CCG CCT GGC CCT CCG GGT CCG CCT GGT CCG CCT GGT CCA

G P P G P P G P P G P P G P P G P P G P 560

1681 CCG GGT CCG CCT GGC AGT GGT TAC ATC CCT GAA GCC CCT CGT GAT GGC CAG GCC TAC GTT

P G P P G S G Y I P E A P R D G Q A Y V 580

1741 CGC AAG GAC GGC GAG TGG GTG CTG CTG AGT ACC TTC CTG TAA

R K D G E W V L L S T F L *

**Figure S2.** DNA and amino acid sequences of MBP-collagen. Left numbers are nucleotide positions. Right numbers are amino acid positions. MBP-collagen contains an N-terminal MBP domain (1-1116), a hexahistidine tag (1117-1134, yellow), a TEV cleavage site (1144-1164, red), a Flag tag (1165-1188, green), a collagen domain (1189-1692, blue) and a C-terminal foldon domain (1699-1779, purple). The 2 inserted Glycine-Proline-Cysteine sequences are underlined. MBP: maltose binding domain. TEV site: Tobacco Etch Virus protease recognition site. Flag tag: an epitope tag for recombinant protein detection and purification.

## II. Recombinant collagen expression and purification

The constructed plasmids were transformed into chemically competent *E. coli* BL21(DE3). Cells were grown overnight at 37 ˚C in LB media supplemented with 30 μg/ml kanamycin. Cultures were diluted 1:100 in fresh LB media containing appropriate selective antibiotics as above and grown for additional 2.5 h until the level of $OD_{600nm}$ was 0.6-0.8. Protein expression was induced by

isopropyl-D-thiogalactopyranoside (IPTG) at a final concentration of 0.2 μM overnight at 15 ˚C. Bacteria were lysed by sonication in buffer containing 50 mM Tris-HCl pH 7.4, 150 mM NaCl, 20 mM imidazole, 1 mM Dithiothreitol (DTT), 1 mM PMSF, protease inhibitor cocktail (Calbiochem), 1.5 μg/ml DNase I and 1.5 μg/ml lysozyme. Lysed cultures were centrifuged at 4 ˚C for 40 min at 50 000 g. The protein in the supernatant was purified by nickel affinity column (HisTrap™ HP) and gel filtration column (HiLoad 16/60 Superdex™ 200 pregrade) on a fast protein liquid chromatography system (FPLC, GE Healthcare). The final gel filtration buffer contains 50 mM Tris HCl, 150 mM NaCl and 1 mM DTT (pH 7.4). DTT was added to prevent collagen aggregation by intermolecular disulfide bonds formation. SDS-PAGE electrophoresis was performed. Samples were either non-heated or heated at 90 ℃ for 5 min in a loading buffer containing β-mercaptoethanol (β-me). Monoclonal Anti-polyHistidine peroxidase conjugate (Sigma, A7058) was used in western blot analysis following the manufacturer's protocol. Detection was performed using an enhanced chemiluminescence system (Western Lightning Plus-ECL, PerkinElmer). Fractions containing MBP-collagen were pooled and concentrated using Amicon Ultra-15 column. Amino acids sequence was confirmed by Liquid chromatography tandem-mass spectrometry (LC-MS/MS).

## III. Characterization of MBP-collagen

Since the elution buffer for MBP-collagen (TBS: 50 mM Tris-Cl, 150 mM NaCl, 1mM DTT) has significant effect on the CD spectra at low wavelength (below 210 nm), TBS buffer was exchanged to water using Amicon Ultra-15 device (30K, Millipore) before CD measurement. Circular Dichroism Spectroscopy (CD) spectra were recorded using an Applied Photophysics–Chirascan spectrometer. Collagen sample was buffer exchanged into pure water before CD measurements. Data were collected in 2 nm increments with a 3 s averaging time, 1 nm bandpass, and 0.1 cm pathlength. The sample was heated from 20 ℃ to 90 ℃ at 5 ℃ increments (heating rate 1 ℃/min). The ellipticity (mdeg) was monitored with a 5 s averaging time, 1 nm bandpass, and 0.1 cm path-length.

## IV. PCR and *in vitro* transcription

A pair of primers was used for PCR amplification of the eGFP gene under regulation of T7 promoter using pIVEX-GFP plasmid[6] as a template (GFP-F and GFP-R, Table 1). The reverse

primer is located upstream to the transcription terminator resulting in run-off PCR fragment lacking a terminator. In this construct the translation stop codon is found 108 bp upstream to the 3' end of mRNA. This distance is sufficient for accommodating a functional ribosome. PCR conditions for eGFP amplification were as follows: A single denaturation step (95 ˚C, 1 min) followed by 35 cycles of: denaturation (95 ˚C, 30 s), annealing (60 ˚C, 1 min), and elongation (72 ˚C, 1.5 min), and a final elongation step of 6 min at 72 ˚C. PCR reactions were performed using 0.5 ml PCR tubes in a final volume of 100 µl including the following components: 40 ng of pIVEX-GFP plasmid, 0.5 µM of each primer (GFP-F and GFP-R), 200 µM of each dNTPs, 1x Phusion buffer and 3.2 U of Phusion DNA polymerase. PCR products were purified by MEGAspin kit (Intron Biotechnology, Daejoen, South Korea) and used for the subsequent transcription reaction. For luciferase gene amplification, similar process was performed except that PT7CFE1-Chis-luciferase plasmid was used as PCR template, while Luc-F and Luc-R were used as forward and reverse primers, respectively (Table 1). The PT7CFE1-Chis-luciferase plasmid was constructed by cloning the luciferase gene into the multiple cloning sites of the plasmid pT7CFE1-Chis (Pierce).

The PCR fragment (43 ng/µl) was in vitro transcribed at 37 ° C for 2.5 hours using T7 RNA polymerase (0.1 mg/ml) in a buffer containing 20 mM $MgCl_2$, 1 mM spermidine, 5 mM DTT, 40 mM Tris pH 7.5, 0.01% Triton X-100, and 4 mM of each NTP. The RNA was purified using the RNeasy Mini Kit (QIAGEN) and quantified by NanoDrop (Thermo Sci., Wilmington, DE).

### V. RNA-Collagen cross-linking by sulfo-SMCC

eGFP or luciferase mRNA was modified with 3'-$NH_2$-ATP (EDA-ATP, Jena Bioscience, Germany) using poly-A polymerase (New England Biolabs) as follows: 100 µl reaction containing 2 µM of eGFP RNA transcript, 0.1 mM of EDA-ATP, 1x reaction buffer and 5 U of *E. coli* Poly(A) Polymerase was incubated for 0.5 hour at 37 ° C. Then the 3'-end amine modified RNA (3'-$NH_2$-RNA) was purified using the RNeasy Mini Kit (QIAGEN) and eluted with nuclease-free water. DTT in the collagen sample was removed by Micro BioSpin P-6 columns (Bio-Rad) before the cross-linking reaction. 10 mg/ml of sulfosuccinimidyl-4-(N-maleimidomethyl) cyclohexane-1-carboxylate (sulfo-SMCC, Thermo Scientific) was dissolved in dimethyl sulphoxide (DMSO, Sigma) and kept frozen (-20 ℃) in a desiccator. Amine modified RNA was conjugated to sulfhydryl-containing MBP-collagen in two steps: the primary amine modification on the RNA is

reacted with the NHS ester moiety of the sulfo-SMCC to attach the bifunctional molecule to the RNA. The SMCC-modified RNA is then conjugated to collagen by addition of the sulfhydryl group of the cysteine residue to the maleimide moiety of sulfo-SMCC. The 3'-$NH_2$-RNA (2.83 μM) was first mixed with 73 mM borate buffer, pH 8.6 and 2.08 mM sulfo-SMCC, vortexed, reacted at 24 ℃ for 1 h, then purified using RNeasy kit to remove excess sulfo-SMCC. The maleimide-modified RNA (442 nM) was mixed with purified MBP-collagen (8.83 μM) in 1 x TBS buffer (50 mM Tris-HCl pH 7.4, 150 mM NaCl, pH 7.4). The molar ratio of collagen:RNA was 20:1. The reaction mixture was incubated at 24 ℃ for 2 hours. RNA, collagen and RNA-collagen complex were mixed with 2×RNA Loading Dye, heated at 65°C for 3 min, cooled in ice, and then loaded onto a 1% agarose gel. Electrophoresis was performed at 100 volts for 30 min in 0.5×TBE buffer (89 mM Tris-borate and 2 mM EDTA, pH 8.3). The gel was stained with 0.5 μg/mL ethidium for 30 min, followed by destaining in water for 30 min with gentle agitation. We also mixed the same amount of unmodified RNA with collagen (RNA-collagen mixture) and incubated in 1 x TBS buffer at 24 ℃ for 2 hours. 1% agarose gel was prepared in TG buffer (25 mM Tris, 192 mM glycine, pH 8.3). Samples were mixed with 2×RNA Loading Dye, heated at 65°C for 3 min, cooled in ice, loaded and then run in TGS buffer (25mM Tris, 192mM glycine, 0.1% SDS, pH8.3) at 100 v for 50 min. The gel was stained with 0.5 g/ml ethidium bromide followed by destaining in water. Bands were visualized via UV trans-illumination using a BioRad Chemilab XRS+ gel-dock system (BioRad, Hercules, CA).

**VI. *In vitro* translation of eGFP and luciferase on collagen scaffolds**

eGFP synthesis on collagen scaffolds was measured using the *E. coli* cell-free translation assay. S12 cell free extract was prepared from *Escherichia coli* strain BL21(DE3) according to previous report.[7] An aqueous solution containing amino acid mix (1 mM for each amino acid), magnesium acetate (20 mM), potassium glutamate (200 mM), *E. coli* tRNA mixture (0.17 mg/mL), DTT (1.8 mM), folinic acid (35 μg/mL), cAMP (0.65 mM), $NH_4OAc$ (28 mM), creatine phosphate (80 mM), HEPES (pH 7.5, 28 mM), 2% w/v PEG-8000, tyrosine (0.04 mg/mL), creatine kinase (0.25 mg/mL) and 17% S12 extract (v/v) were prepared. RNA-collagen or eGFP mRNA (250 ng/μL) was added to this solution commencing the translation reaction in a 96 well plate at 37 ℃. Fluorescence data were collected on a Synergy HT Microplate Reader (BioTek Instruments) ($\lambda_{excite}$ = 485 nm, $\lambda_{emit}$ = 528 nm). Luciferase mRNA and luciferase mRNA-collagen complex were translated in vitro in rabbit reticulocyte lysate system

(Promega). 17.5 µl of rabbit reticulocyte lysate was mixed with 1 µg of mRNA, 20 µM amino acid mixture minus cysteine, 20 µM amino acid mixture minus leucine and 0.8 U/µL RNase Inhibitor (Beyotime biotechnology, china). Then the mixture was incubated at 30 ℃ for 1.5 h. Luciferase assays were performed using the Luciferase Assay System (Promega). 25µl of the in vitro translation product was mixed with 100µl of Luciferase Assay Reagent, and then the luminescence intensity was analyzed using a Tecan Infinite 200 Pro (Tecan, Männedorf, Switzerland) microplate reader.

**Table S2.** Luciferase activity assay

| Template of *in vitro* translation reaction | Negative control | Luciferase mRNA-collagen | Free mRNA |
|---|---|---|---|
| Luminescence intensity (RLU) | 38±26 | 15307±4162 | 24997±76 |

RLU: relative light units. All results are representative of three repeated experiments. RLU represent the means ±standard deviations. Negative control sample contained all in vitro translation reaction components except that no mRNA was added.

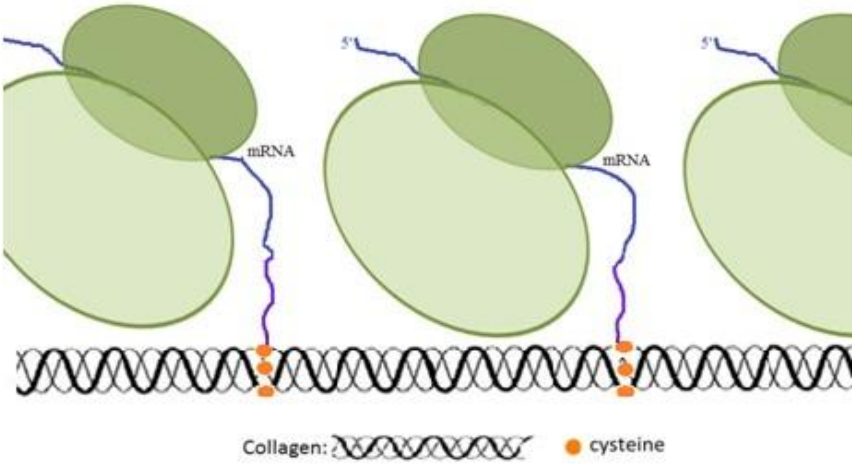


**Figure S3**. Structural representation of the ribosomal biosynthetic machinery on collagen-mRNA platform. Blue strand indicates mRNA, gray/green particles are ribosomes, and orange spots are cysteines on the collagen triple helix strands.

## VII. References.

[1] W. L. DeLano. The PyMOL Molecular Graphics System; Version 1.2r3pre; Schrödinger, LLC, Manheim, Germany, **2008**.

[2] S. Frank, R. A. Kammerer, D. Mechling, T. Schulthess, R. Landwehr, J. Bann, Y. Guo, A. Lustig, H. P. Bächinger, J. Engel, *J. Mol. Biol.* **2001,** *18*, 1081.

[3] Y. Peleg, T. Unger, Application of high-throughput methodologies to the expression of recombinant proteins in E. coli. In *Methods in Molecular Biology*; Kobe, B., Guss, M., Huber, T., Eds.; Humana Press, Totowa, NJ, **2008,** *Vol. 426*, pp. 197-208.

[4] A. Erijman, A. Dantes, R. Bernheim, J. M. Shifman, Y. Peleg, *J. Struct. Biol.* **2011,** *175*, 171.

[5] T. W. Kim, J. W. Keum, I. S. Oh, C. Y. Choi, C. G. Park, D. M. Kim, *J. Biotechnol*. **2006**, *126*, 554.

[6] J. Rogé, J. M. Betton, *Microb Cell Fact*. **2005**, *4*, 18

[7] H. Inoue, H. Nojima, H. Okayama, *Gene* **1990**, *96*, 23.
